# Influence of High-Temperature and Intense Light on the Enzymatic Antioxidant System in Ginger (*Zingiber officinale* Roscoe) Plantlets

**DOI:** 10.3390/metabo13090992

**Published:** 2023-09-04

**Authors:** Min Gong, Dongzhu Jiang, Ran Liu, Shuming Tian, Haitao Xing, Zhiduan Chen, Rujie Shi, Hong-Lei Li

**Affiliations:** 1 College of Biology and Food Engineering, Chongqing Three Gorges University, Chongqing 404100, China; gm137651@gmail.com (M.G.); shumingtian946@gmail.com (S.T.); 2 College of Landscape Architecture and Life Science, Chongqing University of Arts and Sciences, Chongqing 402160, China; 202071714@yangtzeu.edu.cn (D.J.); xinght@cqwu.edu.cn (H.X.); 3 College of Horticulture and Gardening, Yangtze University, Jingzhou 433200, China; 4 Chongqing Tianyuan Agricultural Technology Co., Ltd., Chongqing 402100, China; liuranfy1120@gmail.com; 5 State Key Laboratory of Systematic and Evolutionary Botany, Institute of Botany, Chinese Academy of Sciences, Beijing 100093, China; zhiduan@ibcas.ac.cn

**Keywords:** ginger, malonaldehyde (MDA), superoxide dismutase (SOD), catalase (CAT), environmental stress

## Abstract

Environmental stressors such as high temperature and intense light have been shown to have negative effects on plant growth and productivity. To survive in such conditions, plants activate several stress response mechanisms. The synergistic effect of high-temperature and intense light stress has a significant impact on ginger, leading to reduced ginger production. Nevertheless, how ginger responds to this type of stress is not yet fully understood. In this study, we examined the phenotypic changes, malonaldehyde (MDA) content, and the response of four vital enzymes (superoxide dismutase (SOD), catalase (CAT), lipoxygenase (LOX), and nitrate reductase (NR)) in ginger plants subjected to high-temperature and intense light stress. The findings of this study indicate that ginger is vulnerable to high temperature and intense light stress. This is evident from the noticeable curling, yellowing, and wilting of ginger leaves, as well as a decrease in chlorophyll index and an increase in MDA content. Our investigation confirms that ginger plants activate multiple stress response pathways, including the SOD and CAT antioxidant defenses, and adjust their response over time by switching to different pathways. Additionally, we observe that the expression levels of genes involved in different stress response pathways, such as SOD, CAT, LOX, and NR, are differently regulated under stress conditions. These findings offer avenues to explore the stress mechanisms of ginger in response to high temperature and intense light. They also provide interesting information for the choice of genetic material to use in breeding programs for obtaining ginger genotypes capable of withstanding high temperatures and intense light stress.

## 1. Introduction

Global warming causes frequent climate anomalies, while ecological issues pose significant challenges to agricultural production [1]. Plants are often subjected to various adverse conditions during their growth and development [2]. Temperature and light are two critical factors that impact plant yield [3,4]. High temperature (HT) can trigger the accumulation of reactive oxygen species (ROS), which possess strong oxidizing ability and can damage many biological macromolecules [5]. In normal conditions, plant cells maintain a dynamic balance between the production and elimination of intracellular ROS, which does not pose any harm to plant function [6]. Excessive HT stress can lead to the accumulation of ROS, resulting in decreased enzymatic reaction efficiency, physiological metabolism inactivation, cell death, reduced photosynthetic rate, poor assimilate formation, and even decreased yield and fruit quality [7]. HT conditions are generally accompanied by intense light (IL); this combined stress represents one of the primary limiting factors during the life process of plants. Research has demonstrated that plants are significantly more affected by a combination of HT and IL stress rather than experiencing either HT or IL individually [8].

To combat the harmful effects of ROS, plants have developed a sophisticated antioxidative defense system that includes enzymatic and non-enzymatic antioxidants [9]. These enzymatic antioxidants include SOD, CAT, LOX, and NR [10]. These enzymes work together to scavenge ROS and neutralize them before they can damage cellular components. SOD is active in eliminating excess ROS and superoxide anions in plants [11]. CAT aids the removal of hydrogen peroxide toxicity in plants [12]. However, the action conditions of antioxidant enzymes are generally mild. Severe stress conditions can inactivate them, leading to the breakdown of the enzymatic defense system and the accumulation of hydrogen peroxide and ROS [13]. Studies demonstrate that heat-resistant rice varieties enhance heat stress tolerance by increasing antioxidant enzyme activity and reducing ROS and MDA content under HT stress conditions [14]. LOX is another vital enzyme in plants that catalyzes the production of fatty acid derivatives from phenolic glycerides [15]. This pathway is one of the most vital means of fatty acid oxidation in plants, which is typically activated under stress conditions. LOX is closely linked to plant disease and injury resistance. NR is an oxidoreductase that plays a key role in catalyzing the conversion of nitrate to nitrite [16].

Plants continuously produce ROS in response to stress conditions, resulting in the generation of malonaldehyde (MDA), as activity of antioxidant enzymes and altered levels of transcription of antioxidant enzymes [17,18]. These parameters are commonly employed as indicators of oxidative damage to cell membranes and cells, which in turn, help to assess the HT tolerance of plants [19]. Studies have demonstrated that the cold-inducible gene *RCI3* of *Arabidopsis thaliana* positively regulates salt and drought stresses [20]. Moreover, it has also been revealed that *LOX* genes exhibit differential expression in the roots, stems, leaves, flowers, fruits, and seeds of plants [21]. In tomato, for instance, the 14 *LOX* genes have different expression levels when subjected to four abiotic stresses, including heat, cold, drought, and salt [22]. The expression profile of *SOD* in foxtail millet was investigated in response to drought, salt, and cold treatments using quantitative real-time PCR (qRT-PCR) [23]. Results indicated that each *SOD* gene responded to at least one abiotic stress condition. Meanwhile, Curtis [24] reported a decline in nitrate content in lettuce leaves upon the expression of the *NR* recombinant gene. Conversely, the expression of the *NR* gene was triggered by light, carbohydrate, and NO_3_^−^.

Ginger (*Zingiber officinale* Roscoe) is an herbaceous perennial belonging to the genus *Zingiber* (fam. Zingiberaceae). The rhizomes possess both medicine and culinary properties. Due to its high yield per unit area and substantial economic benefits, ginger is an economically significant crop that warrants widespread cultivation [25]. *Z. officinale* is a shade-loving species [26], therefore it does not tolerate IL. The daily average temperature suitable for ginger growth is 20–28 °C [27]. Strong solar radiation is detrimental for plantlets, especially when combined with an air temperature above 35 °C [28]. Under intense sunlight, ginger plantlets grow poorly, leaf blades do not expand properly, plant forking is delayed, and the transition from vegetative growth to reproductive growth is prolonged. Additionally, the leaf color turns yellowish. Ultimately, these conditions lead to thin ginger plants, low yield, and poor quality [29]. High-temperature-induced damages to ginger have been increasingly common and severe in recent years, leading to substantial losses of production. Notably, during the summer of 2022 in Chongqing (China), prolonged extreme HT periods resulted in pronounced physiological damage to ginger, evident by the widespread effects on crop yield and quality. In July, ginger is at the plantlet stage. During this period, it grows more slowly, with the main focus on stem and root growth. However, few reports are available on the underlying response mechanism of ginger to HT and IL stresses, which subsequently hinders the progression of breeding efforts. Hence, there appears to be an essential need to explore the unresolved response mechanisms associated with HT and IL exposure in ginger crops.

In the present study, we measured the MDA content and enzyme activity of ginger leaves after 4 consecutive days of exposure to natural HT and IL. Furthermore, we employed bioinformatics to analyze the response mechanism of *SOD*, *CAT*, *LOX,* and *NR* genes in ginger to HT and IL stress. We detected several enzyme gene family members and analyzed the various response patterns of enzyme genes under HT and IL stresses.

## 2. Materials and Methods

### 2.1. Plant Materials

Ten rhizomes of *Zingiber officinale* cv. Southwest, also known as Zhugen ginger, were transplanted into ten pots containing sterilized soil (50 cm × 21 cm × 25 cm) on 15 May 2022. Subsequently, they were cultivated in the greenhouse of the College of Landscape Architecture and Life Science, Chongqing University of Arts and Sciences. The cultivation conditions included a temperature of 25 °C, humidity of 75%, light intensity of 200 µE m^−2^ s^−1^ [30], and a photoperiod of 14 h (from 6 am to 8 pm) of light and 10 h of darkness. The light intensity was adjusted using a sun-shading net and LEDs (BN058C LED 11/CW L1200, Signify Luminaires (Shanghai) Co., Ltd. Shanghai, China). This cultivation period lasted for 60 days. Afterward, all ten ginger plantlets were moved to open-air conditions in Chongqing (29°14′21.912″ N, 105°52′14.088″ E), where they were exposed to natural conditions at 8 am from 14 July to 17 July. During this period, we stopped watering the ginger plantlets. The maximum temperature reached peaks above 39 °C and the light intensity reached up to 1920 µE m^−2^ s^−1^ (Table 1). The adult leaves (the third to fifth unfolding leaves from the top) were collected at 8:30 a.m. on the first day as the control group and at 3:00 p.m. on the first, second, third, and fourth days as the test group. In order to carry out subsequent analyses, the plantlets grown in the greenhouse before being moved to open-air conditions were used as the control. Three adult leaves from three randomly selected ginger plantlets were collected, with one leaf chosen from each plant and mixed. The mixed leaves were immediately stored in liquid nitrogen for subsequent determination of enzyme activity and qRT-PCR. Three technical replicates were conducted independently to ensure accuracy and reliability of the results.

### 2.2. Evaluation of Ambient Conditions, Chlorophyll Index, and Plant Phenotypic Changes

To determine the illuminance, air temperature, and leaf temperature around the ginger plant, we employed an illuminometer (Pro’sKit, MT-4617LED-C, Prokit’s Industries Co., LTD., Taiwan, China). To assess the relative chlorophyll index, we utilized a chlorophyll meter (SPAD-502 Plus/DL, Spectrum Technologies, Inc., Aurora, IL, USA). Measurements were taken on leaves from three randomly selected ginger plantlets, ensuring the accuracy and reliability of the data obtained. Plant phenotypic changes were assessed by taking photographs with a Nikon D610 camera (NIKON CORPORATION, Japan) under the following shooting conditions: aperture value: f/7.1, exposure time: 1/125 s, ISO speed: ISO-200, focal length: 28 mm.

### 2.3. Determination of MDA Content and Activity Assay of Enzymes

#### 2.3.1. Determination of MDA Content

The samples were homogenized in an ice bath and the resulting extract volume was used for analysis. Subsequently, the supernatant was collected by centrifuging at 8000× *g* for 10 min at 4 °C. The content of malondialdehyde (MDA) was determined using the thiobarbituric acid (TBA) method [31]. MDA reacts with TBA, forming a red product with a maximum absorption peak at 532 nm. The colorimetry technique allows for estimation of the lipid peroxide content in the sample. Additionally, the absorbance at 600 nm was measured and the difference between the absorbance at 532 nm and 600 nm was used to calculate the MDA content. The samples were then placed on ice for testing. The determination procedure followed the MDA Colorimetric Assay Kit protocol (Chongqing Bonoheng Biotechnology Co., Ltd., Chongqing, China).

MDA content (nmol/g FW) = [ΔA × V ÷ (ε × d) × 10^9^] ÷ (W × V1 ÷ V2)

where V is the total volume of reaction system (L), ε is the molar extinction coefficient of malondialdehyde (L/mol/cm), d is the optical path of cuvette (cm), V1 is the volume of added sample (L), V2 is the volume of extraction solution added (L), W is the sample quantity (g).

#### 2.3.2. Determination of SOD Activity

The sample preparation method is the same as MDA. Superoxide anion (O^2−^) generation occurs through the xanthine and xanthine oxidase reaction system. O^2−^ can reduce nitroblue tetrazolium (NBT) to produce a blue dye, which absorbs at 560 nm. Superoxide dismutase (SOD) can scavenge O^2−^, thereby inhibiting the formation of formazan. The intensity of the blue color in the reaction solution indicates the SOD activity, with a deeper blue color indicating lower activity and a higher SOD activity. The determination procedure followed the Superoxide Dismutase (SOD) Activity Assay Kit (Chongqing Bonoheng Biotechnology Co., Ltd., Chongqing, China).

P = (A1 − A2) ÷ A1 × 100%

SOD activity (U/g FW) = [P ÷ (1 − P) × V] ÷ (W × V1 ÷ V2) × N

where P is the inhibition percentage (U/g), A1 is the absorbance value of the control tube, A2 is the absorbance value of the experimental tube, V is the total volume of reaction (mL), V1 is the sample volume added to the reaction system (mL), V2 is the volume of extract added (mL), W is the sample quantity (g), N is the dilution factor of samples.

#### 2.3.3. Determination of CAT Activity

The sample preparation method is the same as MDA. H_2_O_2_ exhibits a characteristic absorption peak at 240 nm. Catalase (CAT) is capable of decomposing H_2_O_2_, leading to a decrease in absorbance of the reaction solution at 240 nm over time. The CAT activity is calculated based on the rate of change of absorbance. The determination procedure followed the Catalase (CAT) Activity Assay Kit (Chongqing Bonoheng Biotechnology Co., Ltd., Chongqing, China).

CAT(U/g FW) = [ΔA × V ÷ (ε × d) × 10^9^] ÷ (V1 ÷ V2 × W) ÷ T

where V is the total volume of reaction (mL), ε is the H_2_O_2_ molar extinction coefficient, d is the optical path of cuvette (L/mol/cm), V1 is the volume of added sample (mL), V2 is the volume of extraction solution added (mL), T is the reaction time (min), W is the sample quantity (g), 10^9^ is the unit conversion factor, 1 mol is 10^9^ nmol.

#### 2.3.4. Determination of LOX Activity

To prepare the sample for analysis, 0.1 g of the sample was weighed and 1 mL of reagent 1 was added for ice bath homogenization. The supernatant was collected by centrifugation at 16,000× *g* for 20 min at 4 °C. LOX catalyzed the oxidation of linoleic acid, resulting in the formation of an oxidation product with a characteristic absorption peak at 234 nm. The rate of increase in absorbance at 234 nm was measured to calculate the LOX activity. The collected sample was then placed on ice for testing. The determination procedure followed the Plant Lipoxygenase (LOX) Activity Assay Kit (Chongqing Bonoheng Biotechnology Co., Ltd., Chongqing, China).

LOX (U/g FW) = [(A4 − A3) − (A2 − A1)] × V ÷ (W × V1 ÷ V2) ÷ T × 1000

where V is the total volume of reaction system (U/g), V1 is the the supernatant volume of D that was added to the reaction system (mL), W is the sample quantity (g), V2 is the total volume of supernatant (mL), T is the reaction time (min).

#### 2.3.5. Determination of NR Activity

An amount of 0.1 g of the sample was weighed and 1 mL of extracting solution was added for ice bath grinding. The supernatant was collected by centrifugation at 4000× *g* for 10 min at 4 °C. NR catalyzed the reduction of nitrate to nitrite through the reaction: NO_3_¯ + NADH + H^+^ → NO_2_¯ + NAD+ + H_2_O. NADH exhibited a characteristic absorption peak at 340 nm and the change in absorbance value at 340 nm was indicative of the enzyme activity. To induce the sample, it was soaked with an inducer for 2 h. The collected sample was then placed on ice for testing. The determination procedure followed the Nitrate Reductase (NR) Activity Assay Kit (Chongqing Bonoheng Biotechnology Co., Ltd., Chongqing, China).

NR (U/g FW) = [∆A × V ÷ (ε × d) × 10^6^] ÷ (W ÷ V2 × V1) ÷T

where V is the total volume of reaction system (mL), V1 is the volume of added sample (mL), V2 is the volume of extraction solution added (mL), T is the reaction time (min), ε is the molar extinction coefficient of NADH (L/mol/cm), d is the optical path of cuvette (cm), W is the sample quantity (g), 10^6^ is the unit conversion factor, 1 mol is 10^6^ nmol.

### 2.4. Analysis of Enzyme Coding Genes

In order to identify all enzyme coding genes of *SOD*, *CAT*, *LOX*, and *NR* in ginger, we downloaded the HMM model file of each enzyme gene from the Pfam database (http://pfam-legacy.xfam.org/, accessed on 21 December 2022). Based on the HMM file as the search criterion, we executed the Hmm search program in HMMER3.0 on the ginger genome [32]. The candidate genes were judged by using a score value of ≥100 and e-value ≤ e^−10^. To eliminate duplicates, we carried out identification and screening of protein domains via NCBI-CDD (https://www.ncbi.nlm.nih.gov/, accessed on 23 December 2022). As a result, we obtained the protein sequence of each ginger enzyme gene family, followed by the utilization of MEGA7.0 software to perform multiple sequence alignment of each enzyme gene family. Subsequently, the adjacent-joining (NJ) method was employed to construct the phylogenetic tree of enzyme proteins of the ginger gene family and Arabidopsis gene family with the bootstrap value set to 1000; other parameters maintained default [33].

### 2.5. Expression Analysis of Enzyme Coding Genes by qRT-PCR

The study utilized qRT-PCR (qTOWER 2.2, Analytik Jena AG, Germany) to assess the expression of enzyme genes under abiotic-stress conditions. To minimize experimental errors, the materials were subjected to three repeated experiments. All four *ZoCAT* genes were deliberately selected, while for the other three enzymes, four genes were randomly chosen. The primers for qRT-PCR were designed using Primer5 software (http://frodo.wi.mit.edu/, accessed on 29 December 2022) (Table 1). The *ZoTUB2* gene was used as an internal control. The PCR program consisted of an initial denaturation at 95 °C for 30 s, followed by 40 cycles of 95 °C for 10 s and 60 °C for 30 s. Biological triplicates were used for each reaction. The relative expression level of each enzyme gene was calculated using the 2^−ΔΔCT^ method [34].

### 2.6. Statistical Analyses

The statistical analysis was conducted with IBM SPSS Statistics 22. This involved performing a one-way analysis of variance (ANOVA) and species-specific Duncan’s multiple range tests to compare the mean values of different exposure treatments to HT and IL times. Furthermore, Origin Pro 2022 version software was used to generate a column analysis chart using all individual response variable data points. This approach ensured accurate representation of the results and minimized academic plagiarism risks.

## 3. Results

### 3.1. The Changes of Chlorophyll Index under HT and IL Stress

**Figure 1:**
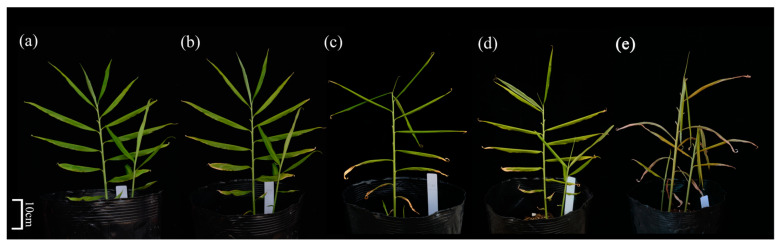
The phenotypic changes of *Zingiber officinale* cv. Southwest before (**a**) and after being exposed to HT and IL for four consecutive days (**b**–**e**).

HT and IL conditions showed significant impact on the growth of *Z. officinale cv.* Southwest, as depicted in Figure 1. With increasing time exposure to HT and IL, ginger leaves displayed prominent signs of curling, yellowing, and even withering. Air temperature, illumination, leaf temperature, and chlorophyll were measured at various time intervals, as reported in Table 2. The temperature and illumination were significantly lower in the control group compared with the experimental group during the four-day period of HT and IL stress. Initially, the chlorophyll index was approximately 29.27 SPAD (soil and plant analyzer development), but it decreased throughout the exposure to HT and IL treatment, reaching its lowest point on the fourth day at 15.9 SPAD.

### 3.2. Malondialdehyde Contents

**Figure 2:**
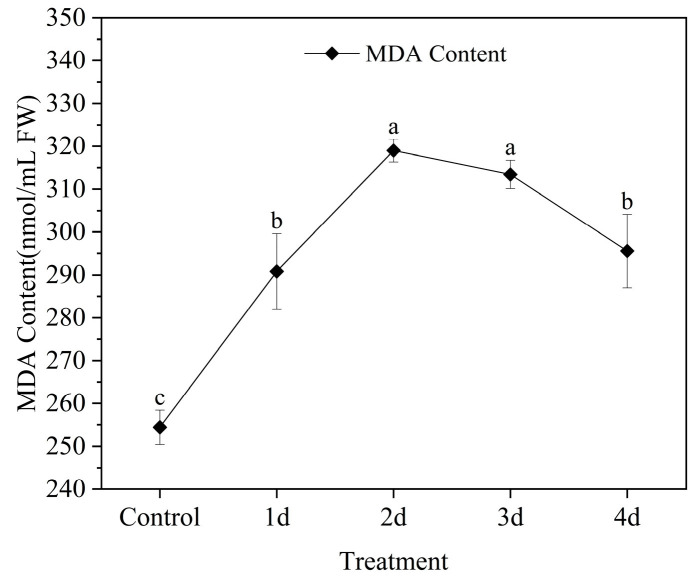
MDA content in leaves of *Z. officinale* under HT and IL treatment for 4 days. Data shown are means ± SD (n = 3). Different lowercase letters in the same group of data indicate significant differences between different treatments (*p* < 0.05).

The MDA content was initially low in the control group but steadily increased from the first day. The highest MDA content was observed on the second day under HT and IL stress (319.00 ± 2.67 nmol/mL FW). From the third day onwards, the MDA content gradually decreased. A significant decrease was observed on the fourth day (Figure 2).

### 3.3. Protective Enzyme Activities

**Figure 3:**
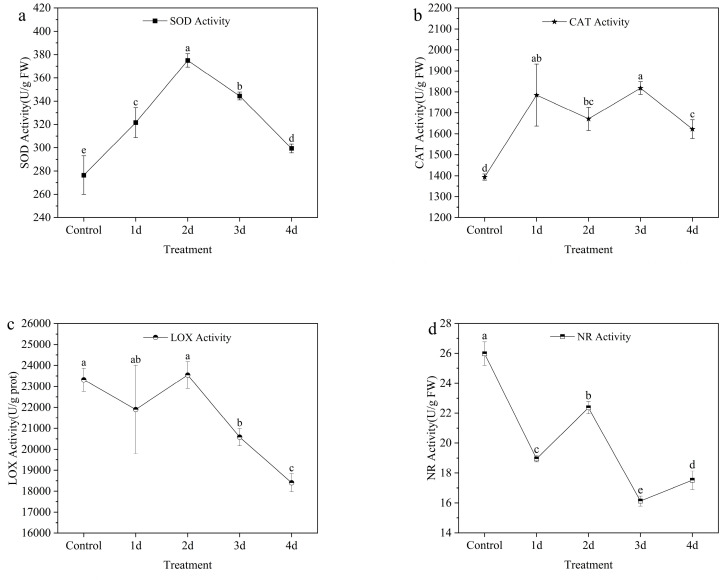
The activities of enzymes (SOD, CAT, LOX, and NR) (**a**–**d**) in leaves of *Z. officinale* under HT and IL for 4 days. Data shown are means ± SD (n = 3). Different lowercase letters in the same group of data indicate significant differences between different treatments (*p* < 0.05).

The activity of SOD exhibited a gradual increase with prolonged exposure to HT and IL. Notably, the highest SOD activity was observed on the second day under HT and IL stress, recording 374.99 ± 5.76 U/g FW (Figure 3a). However, with further exposure to the HT, the SOD activity displayed a gradual decline over time.

Comparing the CAT activity in the control group, there was a significant increase in activity observed on the first day. There was no significant change in CAT activity during the four-day HT and IL stress treatment. The highest CAT activity was observed on the third day, recording 1817.96 ± 30.00 U/g FW (Figure 3b). On the fourth day, the CAT activity slightly declined compared with the first and third day.

During the first two days of the HT and IL treatment, no significant difference in LOX activity was observed, remaining stable at approximately 22,921.74 (U/G prot). However, on the third day, the LOX activity showed a significant decrease compared with the second day. The LOX activity declined to its lowest level on the fourth day, as depicted in Figure 3c.

There was a significant reduction in the NR activity observed on the first day under HT and IL stress. The highest NR activity was recorded on the second day after undergoing the HT and IL (22.36 ± 0.41 U/g FW), while the lowest activity was observed on the third day (16.13 ± 0.33 U/g FW) (Figure 3d).

### 3.4. Identification of Enzyme Coding Genes in Ginger

The HMM models of SOD domain (PF00080, PF00081, PF02777), CAT domain (PF00199), LOX domain (PF01477, PF00305), and NR domain (PF00173) were extracted from the ginger genome data using the HMMER program. Sequences with expected values E less than 10^−5^ were removed and their domain was validated with the CD search program. A total of 19 *SOD* gene family members (78–432 aa), 4 *CAT* gene (560–2348 aa) family members, 21 *LOX* gene family members (187–938 aa), and 28 *NR* gene (101–897 aa) family members were identified. These genes were designated as *ZoSOD1-ZoSOD19*, *ZoCAT1-ZoCAT4*, *ZoLOX1-ZoLOX21*, and *ZoNR1-ZoNR28* according to their positional order on the ginger protein sequence.

### 3.5. Phylogeny of the Enzyme Coding Genes

**Figure 4:**
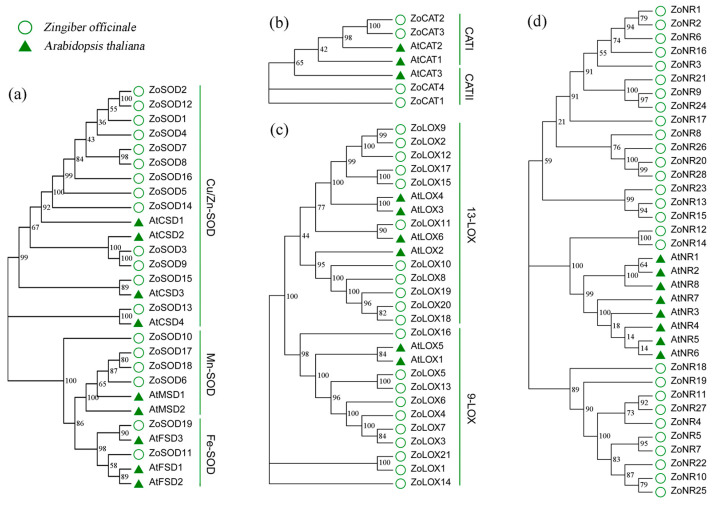
A phylogenetic analysis of enzyme gene families in *Z. officinale*. (**a**) *SOD* family, (**b**) *CAT* family, (**c**) *LOX* family, and (**d**) *NR* family. The round shapes represent the enzyme gene family members of *Z. officinale*, while the triangle shapes represent those of *Arabidopsis thaliana*.

To analyze the phylogenetic relationships between the full-length enzyme gene sequences of *Zingiber officinale* and *Arabidopsis thaliana*, an unrooted phylogenetic tree was constructed based on their alignment. The analysis revealed that the 19 *ZoSODs* could be classified into 3 groups, while the 4 *ZoCATs* could be grouped into 2 groups (I–II). Furthermore, the 21 *ZoLOXs* were clustered into 2 groups and the 28 *ZoNRs* had robust bootstrap value support, indicating their conserved phylogenetic relationships. Based on the phylogenetic analysis, the 28 SOD proteins were classified into 3 groups, namely group I (Cu/ZnSODs), group II (Mn-SODs), and group III (Fe-SODs) (Figure 4a). The seven CAT proteins were categorized into two subfamilies, where subfamily I comprised *ZoCAT2* and *ZoCAT3*, while subfamily II contained *ZoCAT1* and *ZoCAT4* (Figure 4b). Moreover, the 27 LOX proteins could be grouped into two, 9-LOX subtype and 13-LOX subtype, consistent with the types of domains they possess (Figure 4c).

### 3.6. Expression Pattern of Enzyme Genes

**Figure 5:**
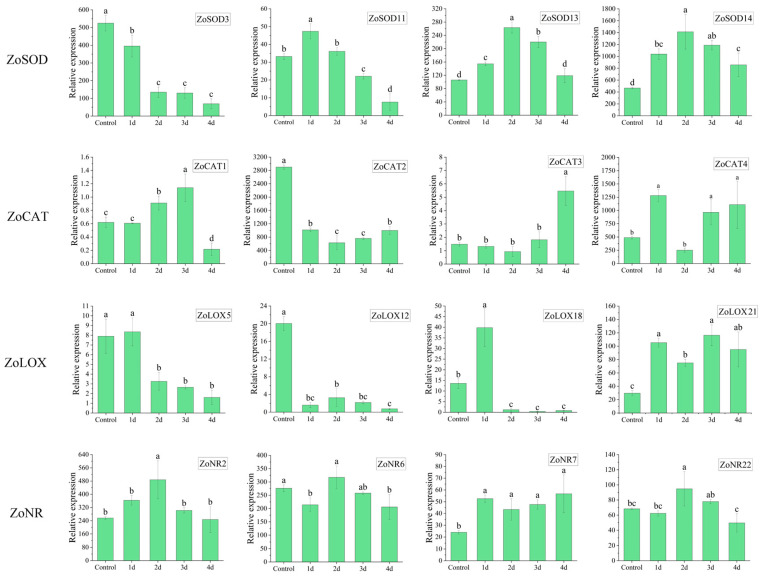
Relative expression of enzyme genes in ginger leaves under HT and IL stress. Data shown are means ± SD (n = 3). Different lowercase letters in the same group of data indicate significant differences between different treatments (*p* < 0.05).

To investigate the involvement of enzyme gene families in adversity, q RT-PCR was employed to analyze the expression of enzyme genes under HT and IL. The results depicted in Figure 5 demonstrated that various enzyme genes displayed different expression patterns under HT and IL stress. On the initial day of HT and IL treatment, there was a notable upregulation in the expression of antioxidant reductase genes such as SOD and CAT, along with a significant downregulation of the oxidoreductase gene LOX, which corresponded with the changes observed in enzyme activity. ZoSOD11, ZoSOD13, and ZoSOD14 exhibited evident upregulation under stress initially, followed by a gradual decrease. The expressions of *ZoCAT1*, *ZoCAT3*, and *ZoCAT4* genes were upregulated. However, *ZoCAT2* displayed a significant downregulation from the initial day. The gene expressions of *ZoLOX5*, *ZoLOX12*, and *ZoLOX18* also showed a significant downward trend, with *ZoLOX21* exhibiting a consistent high level of expression. In contrast, the expressions of *ZoNR2*, *ZoNR6*, and *ZoNR22* genes were initially upregulated on the second day but later downregulated. Conversely, *ZoNR7* showed significant upregulation from the first day and maintained a high level of expression.

## 4. Discussion

The present study demonstrated that *Z. officinale* cv. Southwest is sensitive to HT and IL conditions. This was evidenced by the significant reduction in the chlorophyll index and the increase in MDA content, a marker of oxidative stress, in response to HT and IL stress treatment. These results are consistent with those of previous research indicating that HT and IL stress can cause oxidative damage to plant cells, leading to a decrease in chlorophyll index and an increase in MDA content [35]. The subsequent decrease in MDA may have resulted from the damage caused by continuous high-temperature stress to the intracellular membrane system of ginger cells [36].

In recent years, some research reports have suggested that SOD can protect plants from abiotic and biotic stress, such as heat, cold, drought, and salinity [37,38]. We observed a consistent increase in SOD activity on the first day of HT and IL stress treatment, suggesting the activation of an antioxidant response system in ginger plants. A similar response was also reported in other plant species, such as mung bean (*Vigna radiata* (L.) *R.* Wilczek), wheat (*Triticum aestivum* L.), and tomato (*Solanum lycopersicum* L.), which also showed an increase in SOD activity under HT stress [39,40]. The increase in SOD activity could be attributed to the excessive production of superoxide radicals that are toxic to cells, making defense against oxidative stress important for plant cells. Under various environmental stress conditions, researchers have found that different types of *SOD* genes have different expression patterns. Under stress conditions, *SOD* genes in Arabidopsis show different degrees of differential expression; *FSD1* is a gene that shows significantly increased expression under heat stress [41]. *ZoSOD11* is a direct homologous gene of *AtFSD1* and it shows significant differential expression in ginger under HT and IL stress. In addition, the expression patterns of *ZoSOD13* and *ZoSOD14* are similar to that of *ZoSOD11*, indicating that the function of these genes is related to HT and IL stress. On the other hand, *SOD3* and other genes are not significantly expressed under stress. This is consistent with previous research, such as in Arabidopsis, where the expression of *MnSOD* did not change under oxidative stress conditions, but researchers found that *MnSOD* expression significantly changed under drought stress in wheat and cotton [42,43]. On the second day, SOD activity decreased. This is consistent with Erman’s findings that SOD activity under high temperature stress showed a trend of increasing and then decreasing [44]. This is because, as the stress temperature increases and the duration of stress prolongs, plants continue to increase the production of antioxidant enzymes within their own bodies. However, there comes a point where the plants’ internal regulation cannot adapt quickly enough to the changing external environment, leading to a gradual decrease in antioxidant enzyme levels. Previous studies have reported that under various abiotic stresses, the production of reactive oxygen species (ROS) exceeds the cell’s antioxidant defense capability, resulting in cellular damage [36].

In this study, we observed a notable increase in CAT activity during the first day of the HT and IL stress treatment. This finding aligns with previous research, suggesting that ginger plants activate the CAT pathway in response to stress, similar to rice (*Oryza sativa* L.), which demonstrates a prolonged elevation in CAT activity under HT stress [45]. Liebthal et al., however, reported a decrease in CAT activity in tomato leaves subjected to heat stress [46]. This discrepancy could be attributed to different sensitivity between plant species and diversity in growth conditions that lead to different responses. Comprehensive genomic analyses of the *CAT* family have been widely conducted and three *CAT* genes have been identified in Arabidopsis [47], maize [48], rice [49], and cucumber [50], while we identified four ginger *CAT* genes. The *CAT* genes (*CAT1-3*) in Arabidopsis showed differential expression under salt, cold, heat, and IL stress, but *CAT2* was mainly upregulated, while *CAT3* tended to be downregulated. *CAT2* was the gene with the most significant upregulation under IL; expression also significantly increased under heat stress [41]. In this study, *CAT* genes were all affected by HT and IL stress. Overall, the *ZoCAT1*, *ZoCAT3*, and *ZoCAT4* genes showed upregulation, with some variability in expression patterns under HT and IL stress. This suggests that these genes were involved in activating ROS metabolism. The expression of *ZoCAT2* gene was significantly downregulated, possibly due to some transcription factors inhibiting the expression of *ZoCAT2* gene. Similar reports have been found in Arabidopsis, in which transcription factor *WRKY75* inhibited the expression level of *AtCAT2*, enhanced ROS accumulation, and led to the senescence of Arabidopsis leaves [51].

Our results showed that LOX activity decreased gradually over time, suggesting a redirection of the plant’s response pathway in coping with environmental stresses. This is consistent with earlier findings, where changes in LOX activity have been reported in other plant species, such as *Arabidopsis thaliana* [52]. The lower LOX activity may imply that the plant is mitigating the effects of the stress using an alternate pathway rather than the LOX-dependent pathway. Six types of LOX have been identified in Arabidopsis [53] and *AtLOX2* is required for wound-inducible JA accumulation, but it is unclear if it participates in responses to other stresses [54]. It has been reported that 13-LOX enzymes contribute to responses to abiotic stresses [50,55]. According to phylogenetic analysis, we found that ginger *ZoLOX* genes can also be divided into two subfamilies: 13-LOX and 9-LOX. The expression of the *ZoLOX18* gene belonging to the 13-LOX subfamily, which is close to *AtLOX2*, was significantly upregulated on the first day. Therefore, it is reasonable to speculate that *ZoLOX18* may also respond to HT and IL stress by affecting the JA biosynthesis pathway.

As nitrate is the substrate for the NR reduction process, it directly induces NR activity. NR catalyzes the formation of NO from nitrate or nitrite and NADH or NADPH [56]. NO generated through NR has been identified in sunflower (*Helianthus annuus* L.), spinach (*Spinacia oleracea* L.), and maize [56,57]. Low concentrations of NO and H_2_O_2_ act protectively in the defense response, but high concentrations of NO and H_2_O_2_ can cause serious damage to plant tissues [58,59]. NR activity decreased over time and exhibited a significant reduction in NR activity on the third day of the HT and IL stress treatment. We also found that under IL stress, *NR* gene expression significantly changed and began to rise, reaching its peak on the second day, suggesting a negative effect of at least one of HT and IL on nitrogen assimilation. Ginger plantlets did not receive water during the 4-day exposure to the HT and IL environment. The effects of water deficiency cannot be excluded, although it would not cause death in ginger. It will be interesting to investigate the response of ginger to extreme drought in the future.

## 5. Conclusions

Ginger is sensitive to HT and IL stress, as evidenced by the decrease in chlorophyll index and the increase in MDA content. The present study provides new insights into the antioxidant responses of ginger plants to HT and IL stress. The results suggest that ginger plants activate multiple stress response pathways, including the SOD and CAT antioxidant defense mechanisms, and adjust their response over time by switching to different pathways. Furthermore, we found that the expression levels of genes involved in various stress response pathways, such as *SOD*, *CAT*, *LOX*, and *NR*, were differentially regulated under stress conditions. Our findings highlight the multifaceted nature of ginger plant responses to environmental stress and provide basic information for understanding the antioxidative protection mechanisms of the ginger plant.

## Figures and Tables

**Table 1:** Primer sequences used in qPCR.

Gene Name	Forward Primer (5′ → 3′)	Reverse Primer (5′ → 3′)
ZoSOD3	GTGACCTGGGAAACATCG	GCCAATCTTCCTCCAGCA
ZoSOD11	AAACAGGCGGAACACGAC	CAGTCCCTTCCCATCCAG
ZoSOD13	TTTCTGCTTCTGTTGCCGAGTT	GCTGCTTCCTCGGTCAAA
ZoSOD14	GGCTGTTGCTGTCCTGGGTA	AGGGGAATCTGGCTATCAACA
ZoCAT1	GCTGCTGCGATTCTGTCT	CTTTCGGTTCTCCAGTCG
ZoCAT2	GCACCGTCTAGGACCAAAC	GCTTTCTCACGCCTTCCT
ZoCAT3	ATTCTTGACTTCTCGCACCA	CAGCAGCAATAGAATCATAAAG
ZoCAT4	GTGGGCAGGCTGGTGCTC	TGAGTCCGTCGTAGTGGTTG
ZoLOX5	GCCTACGCCTACTACAACGACC	CACATAGATGTCCAGGCTTAGC
ZoLOX12	GCAAGGTGGAGATGATAACG	TCGGGCGTATCGTATCTG
ZoLOX18	CGAGGCGTTGGAGCAGAAGA	TAATGCCGTCGCAGTTGAT
ZoLOX21	GCCCTGCTGGATGAACCT	ATCGCCCGAGTTGGACCTG
ZoNR2	TGTGAAGGTCTACACCCTGACTGAG	TCCTGGTTGTAGTGCGGTTG
ZoNR6	TCATCCACTGGGAAAGATGC	AATCTGAGGCCCACAGCA
ZoNR7	TTCTGAAGTAACTGAGGGCACA	CTGCCAGTTGCTCCACGA
ZoNR22	GTTCTTTACGGTGCTGGCTTTG	GGTCCACCTGGTCCATAAA
ZoTUB2	GAACATGATGTGTGCTGCCG	ATCTTCAGCCCTTTCGGAGG

**Table 2:** Ambient conditions, leaf temperature, and chlorophyll index of *Zingiber officinale cv*. Southwest under HT and IL.

Treatments	Daily Temperature Range (°C)	Average Ambient Temperature during Measurement (°C)	Leaf Temperature (°C)	Light Intensity (μE m^−2^ s^−1^)	Chlorophyll Index (SPAD)
control	25	30.67 ± 0.57 d	28.95 ± 0.49 d	982.44 ± 78.60 e	29.93 ± 0.85 a
1d	33~41	39.00 ± 0.26 c	34.70 ± 0.63 b	1920.01 ± 6.49 a	29.27 ± 0.57 a
2d	34~40	41.23 ± 0.40 b	33.58 ± 0.70 c	1786.31 ± 10.38 d	24.17 ± 0.31 b
3d	31~39	41.30 ± 0.75 b	35.04 ± 0.59 ab	1792.85 ± 16.23 c	20.53 ± 1.25 c
4d	34~40	42.90 ± 0.66 a	36.04 ± 0.32 a	1810.04 ± 9.26 b	15.90 ± 0.55 d

PS: data shown are means ± SD (n = 3). Different lowercase letters after the same column data indicate significant differences between different treatments (*p* < 0.05).

## Data Availability

The datasets presented in this study are available upon request to the corresponding author. Data is not publicly available due to privacy.
